# Kentucky’s Triumph

**Published:** 1897-09

**Authors:** 


### KENTUCKY'S TRIUMPH.

WHILE many states are absorbed with questions relating to preliminary studies and other preparations for the practice of medicine, it has remained for Kentucky to master one quite as important—namely, that relating to persons unlawfully pretending to be doctors, who are robbing people of their money as well as destroying their health and, in some instances, their lives.

The state board of health of Kentucky, presided over by Dr. Joseph M. Mathews, of Louisville, has pursued a relentless war against all forms of irregular medicine until there is nowhere within the borders of that great commonwealth a single individual engaged in the illegal practice of the healing art. All advertising quacks, itinerant peddlers of nostrums, pretending doctors and every person offering to cure disease in any irregular or unauthorised manner, have been driven from the state. Several cases have been tried in the courts bearing on the various forms of quackery, in all of which the board has been sustained in its prosecutions, until now there is absolutely no doubt about its power in all these cases and its authority is no longer questioned even by the guilty, unless it be to effect delay.

The latest triumph of the board is in the case of a "professor of osteopathy," who practised his legerdemain in Louisville

This form of trickery is seeking recognition quite generally in the midwest and has a few outlying disciples scattered easterly even as far as Buffalo. This kind of deception is little else than mere massage, but differs from the latter in that it pretends to cure joint and other deformities, whereas massage is an aid to scientific medicine, to be used in the discretion of a physician like any other therapeutic measure. In the case recently tried in Louisville it was charged that the osteopath twisted, pulled and flexed the leg and thigh of a little sufferer from hip disease, so that he set up a febrile reaction that necessitated medical treatment. Judge Thompson, before whom the case was tried, gave his opinion that this mountebank had subjected a child laboring under tuberculous disease of the hip-joint to cruel and unnecessary torture, that affected its health and necessitated the employment of a real physician to avert calamitous results. The learned judge further defined the practice of medicine as follows : "Any person who, for compensation, professes to apply any science which relates to the prevention, cure or alleviation of the diseases of the human body, is practising medicine within the meaning of the statute." This concise yet comprehensive definition, which will doubtless serve as a precedent, will include the christian science pretenders—a sect so slippery that it has heretofore evaded every attempted application of the law.

New York, with its splendid system of medical education, guarding the entrance doors with a comprehensive preliminary education, its four years of medical collegiate training and its final state examination for license, has much to be proud of ; but heretofore it has proved itself to be totally incompetent to exclude advertising, itinerant, "consultation free" or other similar quacks from its borders. It may, therefore, bowing humbly before the proud commonwealth of Kentucky, lay its tribute at the feet of a great profession that has done as much as that of any other state or country to elevate the standard of legitimate medicine and make it respected of all men.

